# The effect of maternal diabetes on the Wnt-PCP pathway during embryogenesis as reflected in the developing mouse eye

**DOI:** 10.1242/dmm.017723

**Published:** 2014-12-24

**Authors:** Beatriz López-Escobar, David A. Cano, Anabel Rojas, Beatriz de Felipe, Francisco Palma, José A. Sánchez-Alcázar, Deborah Henderson, Patricia Ybot-González

**Affiliations:** 1 Grupo de Neurodesarrollo, Unidad de Gestión de Pediatría, Instituto de Biomedicina de Sevilla, Hospital Universitario Virgen del Rocío, Centro Superior de Investigaciones Científicas, Universidad de Sevilla, 41013 Seville, Spain.; 2 Unidad de Gestión Clínica de Endocrinología y Nutrición, Instituto de Biomedicina de Sevilla (IBiS), Hospital Universitario Virgen del Rocío/Consejo Superior de Investigaciones Científicas/Universidad de Sevilla, 41013 Sevilla, Spain.; 3 Centro Andaluz de Biología Molecular y Medicina Regenerativa (CABIMER), 41092 Sevilla, Spain.; 4 Unidad de Experimentación animal. Instituto de Biomedicina de Sevilla, Hospital Universitario Virgen del Rocío, Centro Superior de Investigaciones Científicas, Universidad de Sevilla, 41013 Seville, Spain.; 5 Centro Andaluz de Biología del Desarrollo (CABD) 41013 Sevilla, Spain.; 6 Institute of Human Genetics, Newcastle University, Newcastle upon Tyne, NE1 3BZ, UK.

**Keywords:** Diabetes, Wnt-PCP pathway, Daam1, Eye defects, Heart defects, Neural tube defects

## Abstract

Embryopathies that develop as a consequence of maternal diabetes have been studied intensely in both experimental and clinical scenarios. Accordingly, hyperglycaemia has been shown to downregulate the expression of elements in the non-canonical Wnt-PCP pathway, such as the *Dishevelled-associated activator of morphogenesis 1* (*Daam1*) and *Vangl2*. Daam1 is a formin that is essential for actin polymerization and for cytoskeletal reorganization, and it is expressed strongly in certain organs during mouse development, including the eye, neural tube and heart. *Daam1^gt/gt^* and *Daam1^gt/+^* embryos develop ocular defects (anophthalmia or microphthalmia) that are similar to those detected as a result of hyperglycaemia. Indeed, studying the effects of maternal diabetes on the Wnt-PCP pathway demonstrated that there was strong association with the *Daam1* genotype, whereby the embryopathy observed in *Daam1^gt/+^* mutant embryos of diabetic dams was more severe. There was evidence that embryonic exposure to glucose *in vitro* diminishes the expression of genes in the Wnt-PCP pathway, leading to altered cytoskeletal organization, cell shape and cell polarity in the optic vesicle. Hence, the Wnt-PCP pathway appears to influence cell morphology and cell polarity, events that drive the cellular movements required for optic vesicle formation and that, in turn, are required to maintain the fate determination. Here, we demonstrate that the Wnt-PCP pathway is involved in the early stages of mouse eye development and that it is altered by diabetes, provoking the ocular phenotype observed in the affected embryos.

## INTRODUCTION

Embryogenesis involves complex processes that are tightly regulated by different signalling pathways, the activity of which can be altered by genetic and environmental factors. Many such alterations lead to embryonic malformation, which can be enhanced if combined. One important environmental factor that has a strong influence on development is maternal diabetes, making diabetic embryopathy one of the most distressing complications of diabetes. Pre-existing maternal diabetes substantially increases the risk of congenital malformations. Improvements in glycaemic control during pregnancy in the last few decades have greatly reduced the incidence of congenital malformations associated with maternal diabetes, although it still remains a considerable health concern (Evers et al., 2004; Garne et al., 2012; Salbaum and Kappen, 2012; Newham et al., 2013; Barisic et al., 2014; Vinceti et al., 2014; Wei and Loeken, 2014). Indeed, because the prevalence of type 2 diabetes is increasing in the western population, it is becoming more urgent to carry out research into the consequences of diabetic pregnancies in order to establish parameters to prevent or reduce the impact of this condition. The most common forms of diabetic birth defects are those involving the cardiovascular and nervous systems, as well as those affecting other organs (for reviews, see Zabihi and Loeken, 2010; Mitanchez et al., 2014). Other malformations include, to a lesser extent, eye malformations that provoke anophthalmia or microphthalmia (Torchinsky et al., 1997; Machado et al., 2001; Atasay et al., 2002; James et al., 2007; Oyama et al., 2009).

Hyperglycaemia affects the activity of important signalling pathways that are crucial for mouse embryogenesis, including the hypoxia (*Hif1α*), *PDGFRa*, Wnt-β-catenin (*GSK3β*, *β-catenin*) and the Wnt-planar cell polarity (PCP) (*Vangl2*, *Daam1*, *Wnt5a*, etc.) pathways (Pavlinkova et al., 2009). There are two major Wnt pathways: the canonical pathway that stabilizes β-catenin; and the Wnt-PCP pathway that reorganizes the actomyosin cytoskeleton, and that governs cell polarity and alignment during tissue morphogenesis (Chen et al., 2008; for reviews, see Zallen, 2007; Komiya and Habas, 2008). Evolutionary conserved genes, initially identified in *Drosophila*, constitute the core PCP pathway genes that drive cell movement in multiple tissues. These genes encode *Frizzled* (*Fz*) (Vinson et al., 1989; Djiane et al., 2000), *Starry night/Flamingo/Clsr* (Chae et al., 1999; Usui et al., 1999; Curtin et al., 2003), *Van Gogh*/*Strabismus/Van Gogh-like (Vangl)* (Taylor et al., 1998; Wolff and Rubin, 1998; Kibar et al., 2001; Murdoch et al., 2001), *Prickle* (*Pk*) (Gubb et al., 1999; Divecha and Charleston., 1995), *Dishevelled* (*Dsh*) (Perrimon and Mahowald, 1987; Sussman et al., 1994) and *Diego (Dgo)/inversin* (Feiguin et al., 2001; Morgan et al., 1998). The Wnt-PCP pathway is further subdivided into two branches, the JUNK and ROCK (Rho Kinase) signalling pathways, of which the latter appears to be crucial in embryogenic events such as heart or neural tube formation (Ybot-Gonzalez et al., 2007; for reviews, see Brade et al., 2006; Henderson and Chaudhry, 2011).

The Daam1 protein plays a crucial role in the Rho-dependent pathway, associating with Dishevelled (Dsh) (Habas et al., 2001) to activate ROCK and promote migration through the formation of actin stress fibres and focal adhesion maturation (Chihara et al., 1997). Daam1 is a member of the formin protein family that is implicated in actin polymerization and that has been shown to be essential for mammalian heart morphogenesis. Thus, *Daam1^gt/gt^* mice generally exhibit multiple heart defects, including ventricular non-compaction, double outlet right ventricles and ventricular septal defects (Li et al., 2011). These cardiac defects are thought to be induced by altered Wnt-PCP signalling because mice carrying mutations in distinct components of this pathway present similar defects (e.g. *Wnt5a*, *Wnt11*, *Dsh2* and *Vangl2*) (Hamblet et al., 2002; Phillips et al., 2007; Schleiffarth et al., 2007; Zhou et al., 2007).

**TRANSLATIONAL IMPACT**

**Clinical issue**

Diabetic embryopathy is one of the most distressing complications of diabetic pregnancy. Despite considerable improvements in glycaemic control in recent years, the incidence of congenital malformations in diabetic pregnancy is still higher compared to non-diabetic pregnancies. Considering that the prevalence of diabetes, particularly type 2 diabetes, is increasing in the western population, it becomes necessary to advance our understanding of the mechanisms underlying diabetic embryopathy in order to develop strategies to prevent or reduce the impact of this condition.

**Results**

Here, the authors induced diabetes in female mice to investigate the effects of maternal hyperglycaemia on embryogenesis. They found that hyperglycaemia during pregnancy caused an increased incidence in eye malformations, among other defects, and that Wnt-PCP signalling pathway activity was affected in mouse embryos. Interestingly, mice with a mutation in the Wnt-PCP component dishevelled-associated activator of morphogenesis 1 (Daam1, which is essential for cytoskeletal reorganization) display eye malformations similar to those resulting from maternal hyperglycaemia. Furthermore, eye defects in *Daam1* mutant embryos are dramatically worsened by environmental hyperglycaemia, both in *in vivo* and *in vitro* settings. Altogether, these observations suggest that maternal diabetes might affect the Wnt-PCP pathway, which in turn might be detrimental to organogenesis.

**Implication and future directions**

This study points to the Wnt-PCP pathway as an important mediator in the pathogenesis of diabetes-induced eye malformations. It would be interesting to determine whether the Wnt-PCP pathway also mediates other diabetic embryopathies, such as cardiovascular malformations and neural tube defects. The current results, achieved in animal models, need to be validated in human studies. Particularly, it would be interesting to analyze whether the incidence of these adverse pregnancy outcomes correlates with Wnt-PCP activity in the embryos, which would help develop potential therapeutic strategies.

In mice, eye development starts with a bilateral evagination of the diencephalon to generate the optic vesicles on embryonic day (E)8.5. On E9.5, the distal part of the optic vesicle contacts the surface ectoderm, inducing a thickening of this layer that leads to the formation of the lens placode, considered to be the first step in lens development. Subsequently, an evagination of the lens placode forms the lens vesicle, and the optic vesicle gives rise to the optic cup on E10.5, which thereafter starts to differentiate into the neural and pigmented retina on E12.5 (Gilbert, 2000; Graw, 2010; Hägglund et al., 2013). The invagination of the optic vesicle to form the optic cup is a crucial step in the formation of structures like the optic stalk (presumptive optic nerve), ciliary body or iris. However, because this event requires different signalling molecules, such as retinoic acid, Pax2, Pax6 and Wnt proteins, it is a process that is complicated to analyze (Graw, 2010).

Gain- and loss-of-function studies in different vertebrates have shown the Wnt-Frizzled pathways to be involved in co-ordinating crucial processes during ocular development (reviewed by Fuhrmann, 2008). In Xenopus, the Wnt-PCP signalling pathway is essential for the formation and maintenance of the eye field, mediating morphogenetic movements, as well as the crosstalk of intracellular ephrinB1 and PCP signalling that is required for retinal progenitors to move into the eye field (Lee et al., 2006). Indeed, Wnt-PCP signalling genes, such as *Daam1*, *RhoA* or *JNK*, are required downstream of EphrinB1 for cell migration into the eye field in Xenopus (Poliakov and Wilkinson, 2006). Interestingly, overexpression of members of the Wnt-PCP pathway induces ectopic Pax6 expression in the frog (a transcription factor expressed by eye precursors) (Rasmussen et al., 2001; Maurus et al., 2005). In addition, the defective cell alignment and suture formation in the lens of *Vangl2* and *Celsr1* mutant mice (both are genes that participate in the PCP pathway) indicate that this process is regulated by PCP signalling (Sugiyama et al., 2010). However, the influence of Wnt-PCP signalling in mammalian eye development remains unclear.

Maternal hyperglycaemia is known to affect the expression of components of the Wnt-PCP pathways, including Wnt5a, Vangl2, Rock1 and Daam1 in mouse embryos (Pavlinkova et al., 2009), suggesting that abnormal Wnt-PCP signalling might underlie diabetic embryopathies. To explore the role of Wnt-PCP in diabetic embryopathies, we have examined the consequences of simultaneously inactivating the PCP pathway and inducing hyperglycaemia during pregnancy.

## RESULTS

### Effects of maternal diabetes on the development of C57Bl/6 embryos

We first analyzed the effect of maternal diabetes on PCP in the widely used rodent model of multiple low dose streptozotocin (STZ)-induced hyperglycaemia (50 mg/kg of body weight). STZ is a toxic glucose analogue that specifically destroys pancreatic β-cells in rodents (Giroix et al., 1983). Thus, we assessed how diabetes affects organogenesis by analysing the offspring of C57 females to which STZ was administered before pregnancy. No significant differences in the litter size (*P*=0.455) and the number of resorptions (*P*=0.561) were found in diabetic (administered STZ) and control non-diabetic female mice. However, macroscopic analysis of embryos collected from diabetic dams between E10.5-17 revealed eye malformations to be the most common defect observed. Eye defects were evident in 25.4% (16 out of 63) of the embryos from the diabetic mice analyzed and among them, 25% (four out of 16) involved asymmetric anophthalmia, 43.75% (seven out of 16) complete anophthalmia, whereas the remaining 31.25% developed microphthalmia (five out of 16, affecting either one or both eyes; Table 1).

**Table 1.**
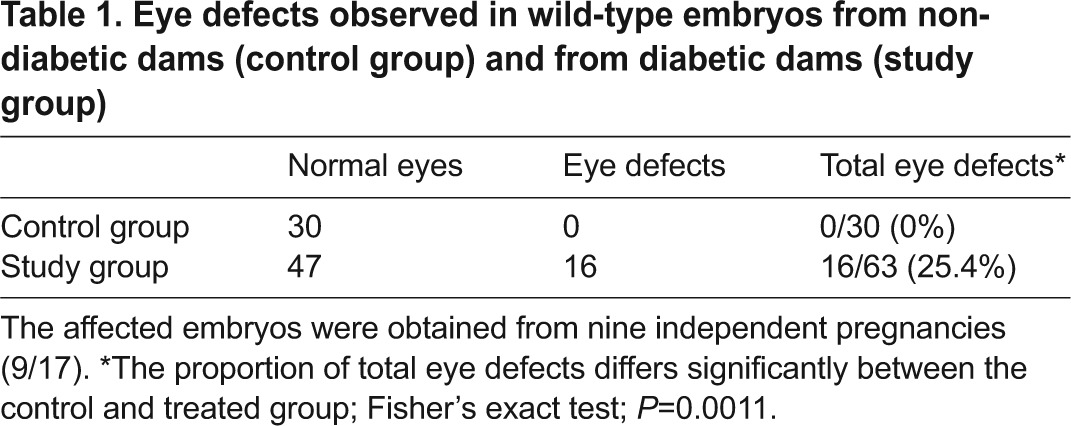
Eye defects observed in wild-type embryos from non-diabetic dams (control group) and from diabetic dams (study group)

**Fig. 1.**
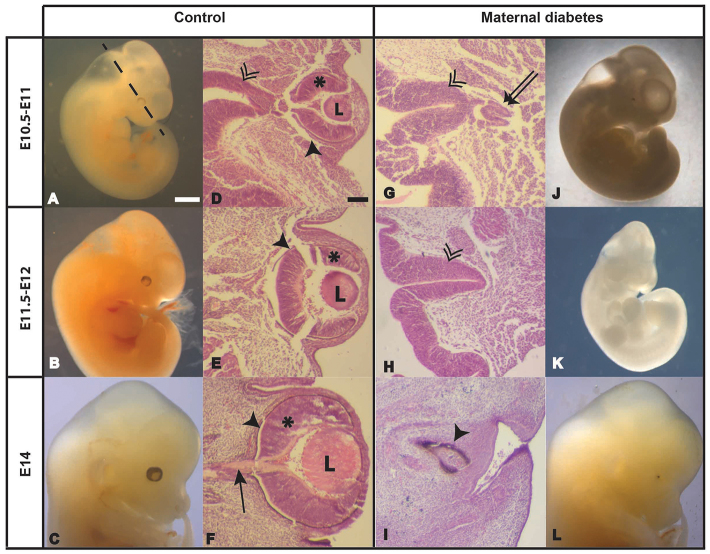
**Effect of maternal diabetes on eye development.** Embryos at E10.5-11 (A,D,G,J), E11.5-12 (B,E,H,K) and E14 (C,F,I,L) from control dams (A–C), and the corresponding transverse paraffin sections stained with haematoxylin and eosin (D–F). Embryos from diabetic dams (J–L) and the corresponding transverse paraffin sections stained with haematoxylin and eosin (G–I). Transverse sections were taken at the level shown by the dashed line in A. The main eye structures are indicated: L, lens; the optic nerve (black arrow); the optic stalk (double arrowhead); rudimentary optic cup (two arrows); the neural retina (asterisk); the retinal pigment epithelium (black arrowhead). Scale bars: 1000 μm (A, also applies to B,C,J–L); 100 μm (D, also applies to E–I). The proportion of total eye defects differs significantly between the control (0/30) and treated group (16/63); Fisher’s exact test; *P*=0.0011.

Histological analysis of the ocular tissue of control embryos at stages E11-14 (Fig. 1A–C) showed a well-developed bi-layered optic cup, in which the inner or neural layer (the future nerve layer of the retina) and the outer layer (the future pigment layer of the retina) could be clearly discerned (Fig. 1D–F). The lens, located in the centre of the optic cup, had a round morphology, with anterior and posterior walls. Within the optic cup, a group of cells appeared in the hyloid cavity, and they were identified as the primitive hyloid plexus of vessels. The thin and long optic stalk could also be distinguished, connecting the optic cup to the brain (Fig. 1F). Macroscopic analysis of embryos at E11 and E14 from diabetic mice that developed microphthalmia revealed gross morphological alterations of the optic cup and presented a smaller and distorted optic cup (Fig. 1G,I,J,L). Also, the optic stalk had not formed but rather, there was a protrusion from the diencephalon that was connected to a very undeveloped optic cup buried in the skull, where no lens was apparent (Fig. 1G). In embryos at E14 from diabetic mice, a well-developed retinal pigment epithelium was evident around the presumptive optic cup, but there was no evidence of other structures (Fig. 1I). Embryos from diabetic dams that developed anophtalmia macroscopically (Fig. 1K) revealed an absence of recognizable optic structures in the histological studies (Fig. 1H).

### Expression of elements of the Wnt-PCP pathway during mouse eye development

**Fig. 2.**
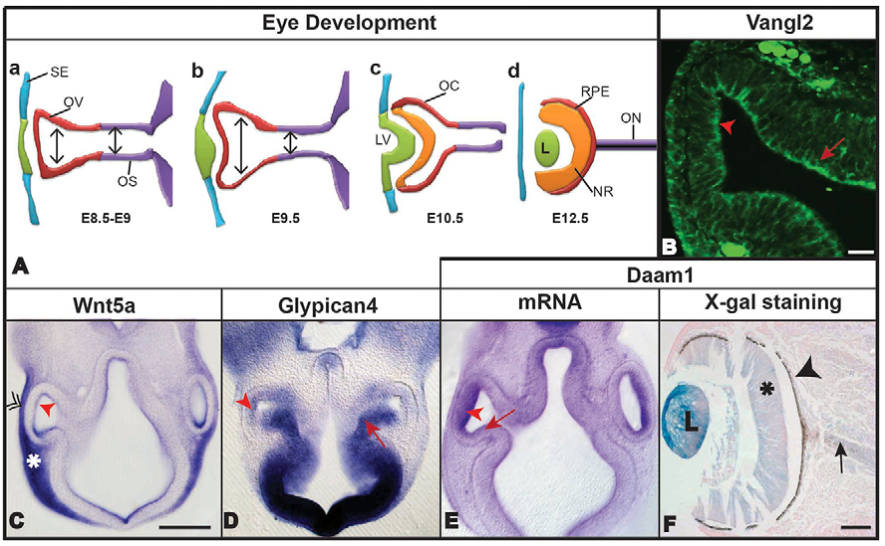
**Wnt-PCP pathway in the developing eye.** Schematic view of the early stages of eye development (A) indicating the main structures: SE, surface ectoderm; OV, optic vesicle; OS, optic stalk; OC, optic cup; LV, lens vesicle; L, lens; RPE, retinal pigmented epithelium; NR, neural retina; ON, optic nerve. Double headed arrows in a and b indicate the luminal diameter. Immunofluorescent localization of Vangl2 in a cryosection from an embryo’s head (B). Whole-mount *in situ* hybridization showing the mRNA distribution in vibratome sections of individual mouse embryos at E9.5 for *Wnt5a* (C), glypican4 (D) and *Daam1* (E). X-gal staining to detect Daam1 in a *Daam1^+/gt^* embryo head at E12.5 (F). Optic vesicle (red arrowhead), optic stalk (red arrow), surface ectoderm (double arrowhead), perioptic vascular plexus (white asterisk), lens (L), neural retina (black asterisk), retinal pigmented epithelium (black arrowhead) and optic nerve (black arrow). Scale bars: 1000 μm (C, also applies to D,E); 100 μm (B,F).

As the defects described above were observed between E11 and E14, they probably originate at earlier stages of development when eye structures start to appear (Fig. 2A). Disruption of early eye development (from E8.5 to E9.5) would disturb the formation of the optic vesicle, thereby affecting the induction of the lens and the formation of the optic cup, defects that were observed at later stages in our study.

The Wnt-PCP pathway is affected by diabetes during pregnancy (Pavlinkova et al., 2009), and it has been implicated in eye development, especially in lens formation (Lee et al., 2006; Chen et al., 2008; Sugiyama et al., 2010; for review, see Poliakov and Wilkinson, 2006), suggesting that abnormal Wnt-PCP signalling might underlie the eye malformations induced by maternal diabetes. Before testing this hypothesis, we examined the temporal and spatial expression of important Wnt-PCP genes during embryonic development. We focused on E9.5, a developmental stage at which key elements in early eye development can be detected (when optic vesicle evagination and lens induction occurs) (Fig. 2A).

Transcripts for *Wnt5a*, a Frizzled receptor binding protein exclusive to the PCP pathway, were detected in the lens placode, which was clearly differentiated from the surface ectoderm and perioptic vascular plexus (Fig. 2C). We also examined the expression of the glypican4 gene, a member of a family of cell surface heparan sulphate proteoglycans that interact with the Wnt ligands Wnt5a and Wnt11 and that is a positive regulator of the PCP pathway during gastrulation in Xenopus (Ohkawara et al., 2003). Glypican4 mRNA accumulated strongly in the neuroepithelium of the future telencephalon, in the optic stalk and in the optic cup (Fig. 2D). Vangl2 is a transmembrane protein that belongs to the PCP core and, although transcripts encoding *Vangl2* could not be detected by *in situ* hybridization, this protein was clearly located in the apical side of the optic vesicle (Fig. 2B), indicating that it would be active in these cells (Gao et al., 2011; Montcouquiol et al., 2006). Daam1, a formin implicated in the non-canonical Wnt pathway that binds to Dsh and RhoA, was also detected in the optic vesicle, expressed strongly in the dorsal region and in the lens placode. *Daam1* expression was also observed in the optic stalk, as well as in the neuroepithelium of the future diencephalon (Fig. 2E). Daam1 is involved in Xenopus eye development (Lee et al., 2006) and, moreover, it is downregulated as a consequence of maternal diabetes (Pavlinkova et al., 2009). Considering these results and the interesting expression pattern of *Daam1* in the developing eye at E9.5, we decided to analyze *Daam1* expression at later stages of development. Accordingly, Daam1 protein was detected in the lens of embryos at E12.5, especially in the cuboidal epithelium of the capsule, in the optic nerve and in both layers of retina – the nerve layer and the pigment layer (Fig. 2F).

### Eye defects in a gene trap mutant of *Daam1*

**Fig. 3.**
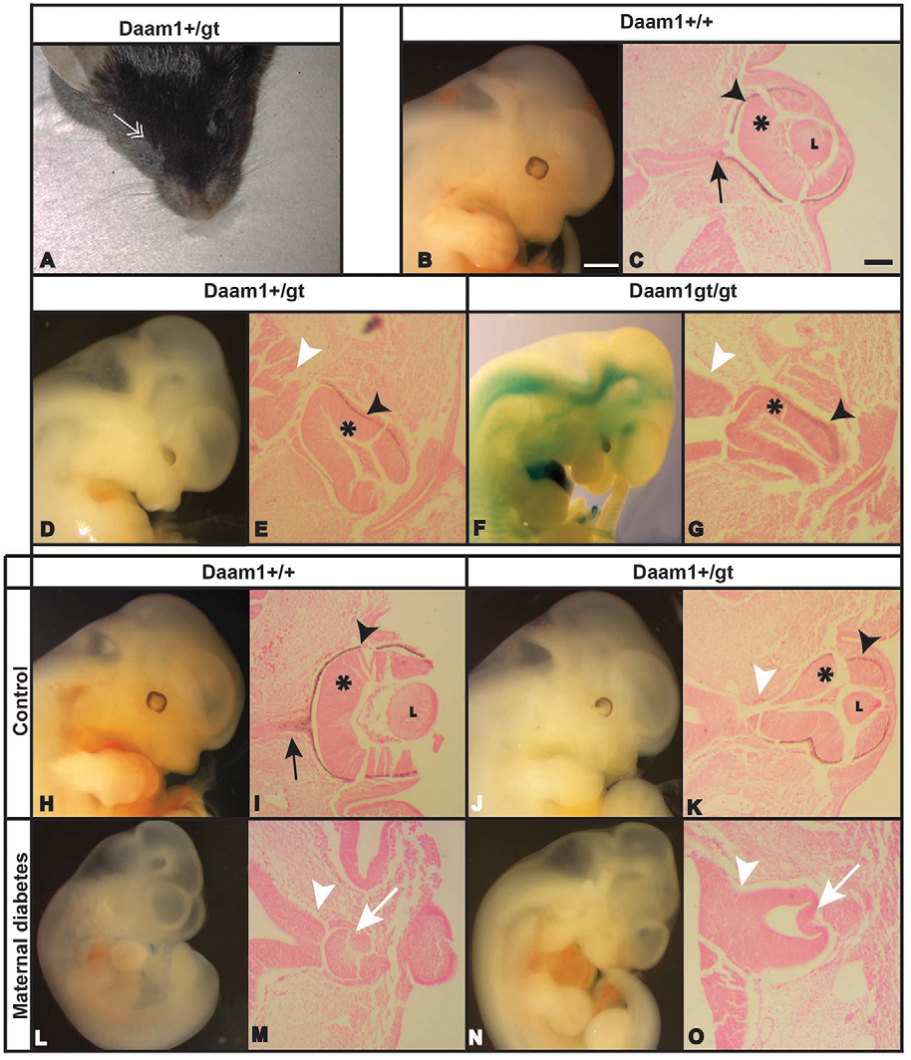
**Ocular defects produced by *Daam1* mutation and in combination with diabetes.** Adult *Daam1^+/gt^* mutant with an eye defect indicated by the white double arrow (A). At E12.5, *Daam1^+/+^* embryo with a normal eye (B), *Daam1^+/gt^* embryo with microphthalmia (D) and a *Daam1^gt/gt^* embryo with microphthalmia (F). Corresponding transverse paraffin sections stained with haematoxylin and eosin (C,E,G). The proportion of total eye defects differs significantly between *Daam1^+/+^* (adults, 0/64; embryos, 0/43) and *Daam^+/gt^/Daam1^gt/gt^* mice (adult *Daam1^+/gt^*, 3/42; *Daam1^+/gt^* embryos, 2/66; *Daam1^gt/gt^* embryos, 1/19; Fisher’s exact test, *P*=0.033). At E12, *Daam1^+/+^* embryo from a control dam with normal eyes (H). At E12, *Daam1^+/gt^* embryo from a control dam with microphthalmia (J). At E11, *Daam1^+/+^* embryo exposed to maternal diabetes with anophthalmia (L). At E12, *Daam1^+/gt^* embryo exposed to maternal diabetes with anophthalmia (N). The corresponding transverse paraffin sections stained with haematoxylin and eosin (I,K,M,O). The main eye structures are indicated: L, Lens; optic nerve (black arrow); optic stalk (white arrowhead); optic cup (white arrow); neural retina (asterisk); retinal pigmented epithelium (black arrowhead). Scale bars: 1000 μm (B, also applies to D,F,H,J,L,N); 100 μm (C, also applies to E,G,I,K,M,O).

Because the PCP pathway appears to participate in eye organogenesis, we assessed its role in eye development by analyzing *Daam1* mutant embryos. For this purpose, we used the *Daam1* gene trap mouse strain (Baygenomics) in which *Daam1* expression is knocked down rather than completely knocked out, as shown by real time (RT)-PCR analyses (data not shown). Notably, eye defects could occasionally be detected during the establishment of this mouse line in *Daam1^+/gt^* animals (three out of 42 *Daam1^+/gt^* mice; 0 out of 43 *Daam1^+/+^* mice; three affected litters out of 14) (Table 2; Fig. 3A). We further analyzed eye formation from E10.5 to E12.5, the earliest stages at which recognizable eye structures can be detected and, thus, when any impairment in optic cup evagination or in lens formation can be seen. Although the wild-type embryos did not present any malformations (Fig. 3B), the gross morphology of the *Daam1^+/gt^* embryos was, generally, not substantially affected, as reported previously (Li et al., 2011), with eye defects in two out of 66 *Daam1^+/gt^* embryos (from two different litters) – one with asymmetric anophthalmia (only one eye affected) and one with microphthalmia (microphthalmia, Fig. 3D). Only one out of 19 *Daam1^gt/gt^* embryos developed an eye defect (Fig. 3F), which was detected in an underdeveloped *Daam1^gt/gt^* embryo when compared with their wild-type and heterozygous littermates (none with eye defects, Table 2). These data indicate that *Daam1* knockdown might interfere with eye formation resulting in major eye abnormalities.

**Table 2.**
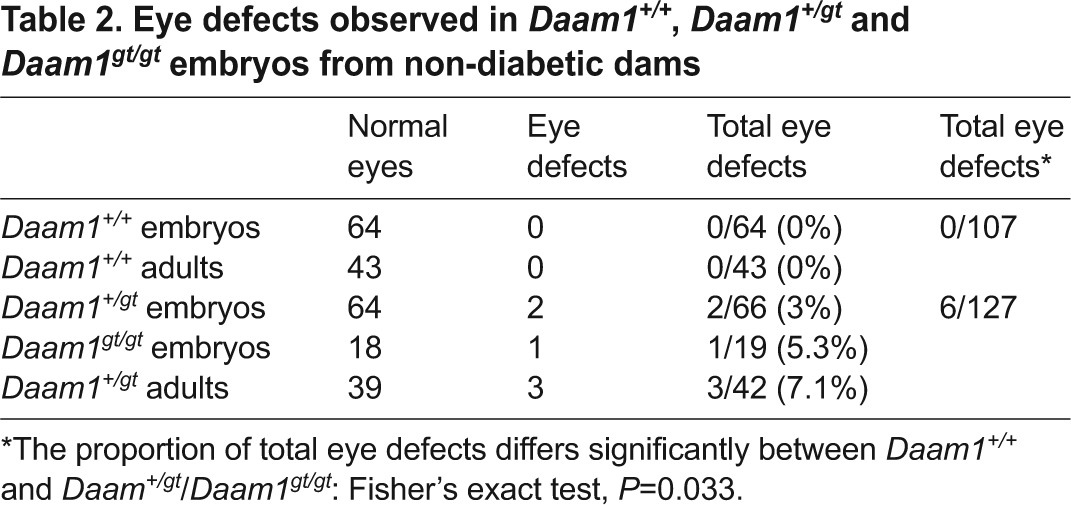
Eye defects observed in *Daam1^+/+^*, *Daam1^+/gt^* and *Daam1^gt/gt^* embryos from non-diabetic dams

In the histological analysis, a dysmorphic optic cup was evident in the *Daam1^+/gt^* and *Daam1^gt/gt^* affected embryos, with no discernible lens, which appears to be replaced by mesenchymal tissue (Fig. 3E,G, respectively, compared with *Daam1^+/+^* embryos Fig. 3C). The optic stalk did not form an optic nerve, which remained thick and short, maintaining the optic cup structure close to the diencephalon. A thickened area appeared at the surface ectoderm where the lens should invaginate but lacked any tissue organization or the formation of a lens structure. In addition, the future eyelid seemed to cover the ventral part of the external side of the eye. These characteristic alterations were similar in both heterozygous and homozygous embryos, although homozygous embryos presented more severe malformations of the eye (Fig. 3E,G). The eye defects observed in the *Daam1* mutant (*Daam1^+/gt^* and *Daam1^gt/gt^*) embryos resembled those found in embryos that had been exposed to maternal diabetes, which prompted us to analyze the combination of these two factors in the context of eye development.

### Ocular defects in *Daam1* mutant embryos at E11.5-12.5 from diabetic dams

To study the interaction between diabetes and the Wnt-PCP pathway during eye development, we analyzed embryos (E11.5-12.5) from STZ-induced diabetic *Daam1^+/+^* females (seven dams) crossed with *Daam1^+/gt^* males, comparing them with control embryos from untreated *Daam1^+/+^* females (four dams) crossed with *Daam1^+/gt^* males. As expected, the eyes in all the *Daam1*^+/+^ embryos (*n*=20) from non-diabetic dams were well-formed (Fig. 3H). By contrast, one *Daam1*^+/gt^ embryo from a control untreated dam developed eye defects (Fig. 3J; 9.1%, *n*=11). Eye defects were found in 17.1% of the *Daam1*^+/+^ embryos that had been exposed to maternal diabetes (Fig. 3L; *n*=41), whereas the incidence of eye defects rose to 27.3% in *Daam1^+/gt^* embryos that had been exposed to maternal diabetes (Fig. 3N; *n*=11, Table 3), although this increase in eye defects was not statistically significant. The affected embryos in this study developed a variety of eye malformations, including anophthalmia (Fig. 3L,N) and microphthalmia (Fig. 3J). In some embryos with microphthalmia, the visible eye structure was a heavily pigmented region with malformed retinal structures (Fig. 3J). In histological studies, *Daam1^+/gt^* embryos of diabetic dams that developed anophthalmia presented a very rudimentary eye, with no structures recognizable as the optic nerve or optic cup (Fig. 3O). These defects were similar to those described in either *Daam1^+/gt^* embryos of control dams (Fig. 3E,K) or *Daam1^+/+^* embryos of diabetic dams (Fig. 1G and Fig. 3M), albeit much more severe. Moreover, in these embryos, the optic stalk appeared as a thick, short structure that was hardly separated from the diencephalons. At the end of this structure, an invagination emerged as a very rudimentary optic vesicle (Fig. 3O). The most severe phenotype observed in *Daam1^+/gt^* embryos of diabetic dams indicated that an interaction occurred between these two risk factors in terms of the induction of eye defects. The fact that the embryopathy induced by maternal diabetes appeared to display certain synergism with the genotype at the *Daam1* locus suggested that the maternal diabetic state affects the non-canonical Wnt pathway, with the *Daam1* mutation exaggerating the diabetes-induced eye abnormalities.

**Table 3.**
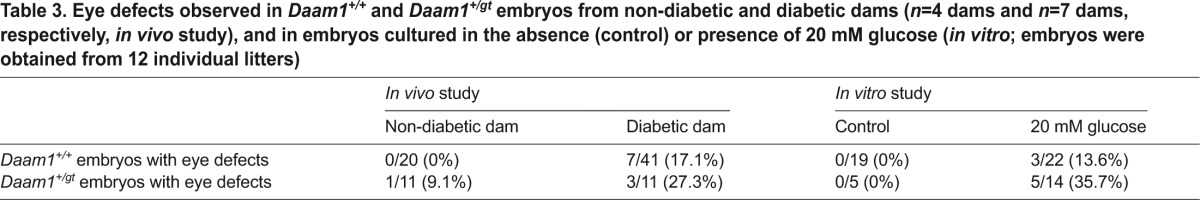
Eye defects observed in *Daam1^+/+^* and *Daam1^+/gt^* embryos from non-diabetic and diabetic dams (*n*=4 dams and *n*=7 dams, respectively, *in vivo* study), and in embryos cultured in the absence (control) or presence of 20 mM glucose (*in vitro*; embryos were obtained from 12 individual litters)

Cardiovascular malformations [CVMs; such as ventral septal defects (VSD)] or the transposition of main arteries, such as the double outlet right ventricle (DORV), and neural tube defects (NTDs) are among the most common embryopathies observed in diabetic pregnancies (Kumar et al., 2007; Morgan et al., 2008; Garne et al., 2012; Fine et al., 1999; Oyama et al., 2009), and they also develop in mutants of the Wnt-PCP pathway (Li et al., 2011; for review, see Henderson and Chaudhry, 2011). Here, CVMs were detected in four out of five *Daam1^+/+^* (two out of five VSD and two out of five DORV) and all four *Daam1^+/gt^* (one out of four with VSD and three out of four with DORV) randomly selected embryos from diabetic dams, all of which were clearly evident in the histological analysis of embryos at E12-17 (supplementary material Fig. S1). Likewise, 29% (*n*=31) of *Daam1^+/+^* and 15.4% (*n*=13) of *Daam1^+/gt^* embryos from diabetic dams developed NTDs, which were not detected in embryos from control dams. These NTDs included a variety of defects – exencephaly (eight *Daam1^+/+^* embryos and one *Daam1^+/gt^* embryo), spina bifida (one *Daam1^+/+^* embryo) and craniorachichisis (one *Daam1^+/gt^* embryo).

### Induction of eye defects in embryos exposed to high levels of glucose *in vitro*

To further study the synergism between decreased PCP signalling and elevated glucose, we turned to *in vitro* models. The growth of embryos in culture allows experimental conditions to be more tightly controlled, ensuring that all the embryos were exposed to the same glucose concentration within a specific developmental window and, in addition, for a limited period of time. Furthermore, embryo culture eliminated any possible toxic or teratogenic effects of STZ administration on embryonic development. In an initial pilot study, toxicity was observed (lack of heart beating and yolk sac circulation) when the cultured embryos were exposed to the highest dose tested (30 mM), whereas at 10 mM and 20 mM, no such toxicity was evident. Accordingly, we chose to assess the effects of administering 20 mM glucose.

**Fig. 4.**
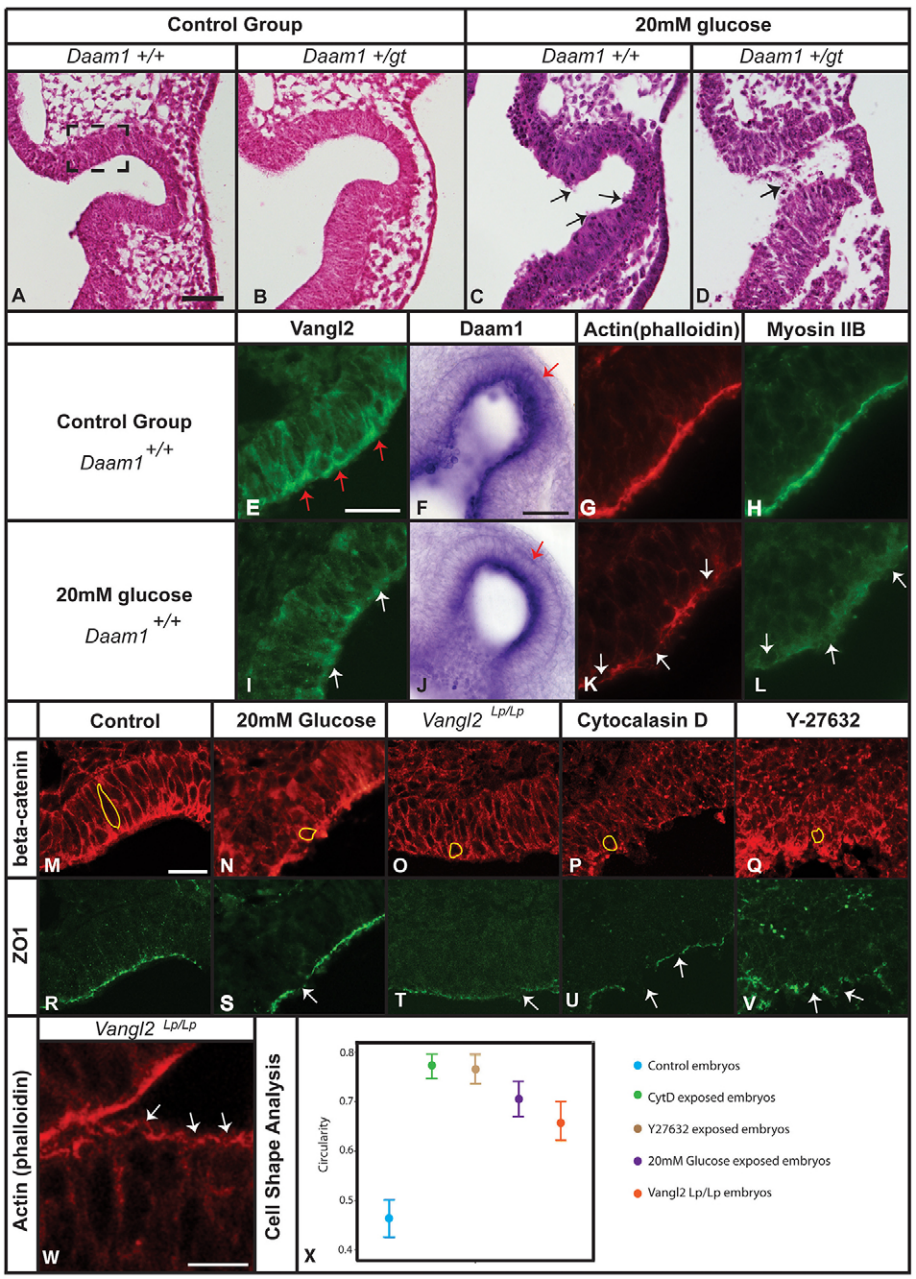
**Histological and immunohistochemical analysis of the developing eye in embryos exposed to glucose *in vitro*.** Control embryos (A,B,E–H,M,R); embryos exposed to 20 mM glucose (C,D,I–L,N,S); *Vangl2^Lp/Lp^* embryos (O,T,W); embryos cultured with cytochalasin-D (P,U) and Y-27632 (Q,V). Detail of the optic vesicle in transverse sections of *Daam1^+/+^* (A,C) and *Daam1^+/gt^* embryos (B,D) stained with haematoxylin and eosin; Vangl2 expression (E,I); *Daam1* mRNA expression (F,J); actin staining using phalloidin (G,K,W); myosin (H,L). Cellular organization of the optic vesicles immunolabelled for β-catenin (M–Q) and ZO1 (R–V). Average circularity of cells along the apical surface (X), where the numbers closer to 1.0 reflect roundness and those closer to 0.0 reflect an increasing deviation from a perfect circle. Global comparison, *P*<0.0001 according to a H-Kruskal–Wallis test and multiple comparisons, *P*<0.0001 using a U-Mann–Whitney test attending to the Bonferroni correction; error bars indicate s.d. Black arrows in C,D indicate unattached cells in the optic vesicle space. The black dashed square in A represents the area of this section shown at higher magnification in E,G–I,K–W. White arrows in panels I,K,L,S–W indicate discontinuity of immunostaining along the apical side of the tissue. Red arrows in E indicate the presence of Vangl2 in the apical side of the optic vesicle, and in F,J indicate *Daam1* expression in the distal side of the optic vesicle. Individual cells have been traced (yellow) in M–Q. Scale bars: 100 μm (A, also applies to B–D); 25 μm (E, also applies to G–I,K,L); 50 μm (F, also applies to J); 25 μm (M, also applies to N–V); 12.5 μm (W).

Embryos at E9 were exposed *in vitro* to 20 mM glucose or maintained under control conditions (Table 3). Embryos that had been treated with glucose developed eye defects (Fig. 4C,D) that were similar to those observed in embryos from diabetic dams. Thus, although the *Daam1^+/+^* embryos cultured in control conditions developed normally (Fig. 4A; none out of 19), 13.6% (three out of 22) of embryos exposed to high glucose levels developed eye defects (Fig. 4C). More interestingly, eye defects were evident in 35.7% of the *Daam1^+/gt^* embryos maintained in the presence of 20 mM glucose (Fig. 4D; five out of 14). *Daam1^+/gt^* embryos did not develop any ocular malformations when cultured in control conditions (Fig. 4B; none out of five). In these experiments, it was not possible to study the *Daam1^gt/gt^* embryos obtained owing to the developmental delay associated with this genotype.

The embryos cultured *in vitro* were classified according to their morphology and thus, in a normal eye at E9.5 (i) the optic vesicle was separated from the diencephalon; (ii) the luminal diameter of the optic vesicle had begun to increase, whereas the luminal diameter of the optic stalk had narrowed progressively (Fig. 2, step b in panel A); and (iii) both the optic stalk or optic vesicle presented a well-organized epithelium at the cellular level (Fig. 4A). Embryos with fewer than two of these characteristics were considered to have deformed eyes. Histological analysis of embryos showed well-established optic vesicles in the *Daam1^+/+^* embryos and the *Daam1^+/gt^* embryos cultured in control conditions (Fig. 4A,B), although a small percentage of embryos in the latter group presented a slight delay in the development of one eye with respect to the other as confirmed by the histological analyses. By contrast, histological analysis of *Daam1^+/+^* embryos that had been exposed to 20 mM glucose demonstrated clear morphological defects in the formation of the optic vesicle, showing an evident disorganization of the apical side of the neuroepithelium with some unattached cells (Fig. 4C). In addition, these embryos had round and small eye invaginations when compared with the optic vesicle of control *Daam1^+/+^* embryos (Fig. 4A,C). The *Daam1^+/gt^* embryos that had been exposed to 20 mM glucose had a more exacerbated phenotype with an underdeveloped optic stalk, showing a disorganized optic vesicle epithelium with ectopic cells in the lumen (Fig. 4D).

### Exposure to high levels of glucose *in vitro* impairs Wnt-PCP signalling and alters the organization of the cytoskeleton

The well-structured optic vesicles in the control embryos contrasted with the severe disorganization of this structure when embryos were exposed to glucose (Fig. 4A–D). Disruption of the PCP pathway provokes cytoskeletal disorganization, with a consequent loss of cell polarity and tissue malformation (reviewed by Simons and Mlodzik, 2008). To determine whether elevated glucose levels induce eye malformations through abnormal PCP signalling, we measured the expression of the PCP genes *Daam1* and *Vangl2*, as well as that of the cytoskeletal components, myosin and actin in embryos cultured in high glucose.

In embryos cultured under control conditions, Vangl2 was concentrated in the apical domain of the optic vesicle cells, indicating that the Wnt-PCP pathway was active in these cells (Fig. 4E). In stark contrast, Vangl2 was diffusely distributed in the optic vesicle cells of embryos that had been exposed to a high glucose concentration, and its expression was markedly weaker in these embryos (compare panels E and I in Fig. 4). These results suggest that the activity of the Wnt-PCP pathway was reduced under diabetic conditions. *Daam1* mRNA expression in the distal side of the optic vesicle was also diminished in embryos that had been exposed to glucose compared with that of the control embryos (compare panels F and J in Fig. 4). Indeed, RT-PCR analyses of the mRNA of cultured embryos at E9 that had been exposed to glucose for only 7 h showed a trend, albeit not one that was significant (*Daam1* expression *P*=0.142; *Vangl2* expression *P*=0.086), towards weaker *Daam1* and *Vangl2* expression. In three of the five embryos that had been exposed to a high concentration of glucose, there was a clear decrease in *Daam1* expression (average fold-change=0.32) with respect to the control embryos (*n*=4, average fold-change=3.14), whereas the other two embryos that received glucose were less severely affected (average fold-change=2.27; supplementary material Fig. S2A). A small decrease in expression was observed for *Vangl2* in the three affected embryos (average fold-change=0.98) when compared with that of the control embryos (*n*=4, average fold-change=2.14), and again the reduction in *Vangl2* expression was weaker in the two remaining embryos that had been exposed to glucose (average fold-change=1.37; supplementary material Fig. S2B).

To investigate the effect of glucose on cytoskeletal organization, we assessed the distribution of actin and myosin by using immunofluorescence. MyosinIIB and F-actin were localized in the inner part of the optic vesicle of embryos cultured under control conditions, forming the continuous actomyosin cable. However, this cable was disrupted and clearly disorganised, presenting gaps along the apical side of the tissue, in *Daam1^+/+^* embryos that had been exposed to glucose (compare panel G with panel K, and panel H with panel L in Fig. 4).

### Eye defects in embryos with an affected Wnt-PCP pathway

We examined the morphological alterations to the optic vesicle in embryos that had been exposed to glucose in more detail by performing immunofluorescent staining for β-catenin. In the control embryos, the cells of the optic vesicles were elongated along the apical-basal axis, and they had a narrow apical surface in the medial-lateral axis, probably driven by apical constriction (Fig. 4M). By contrast, the cells were rounder at the apical side of the epithelium in embryos that had been exposed to glucose (Fig. 4N). The distribution of the ZO1 tight junction protein (an apical marker) in the cells of the vesicle was strongly disrupted in embryos that had been exposed to glucose (compare panels S and R in Fig. 4). Altogether, these observations suggest that the polarization of the optic vesicle cells is impaired in glucose-treated embryos.

For the last part of this study, we decided to verify that the disruption in cell morphology and cell shape observed in glucose-treated embryos could be obtained by just affecting the Wnt-PCP signalling pathway. Because the rate of affected eye phenotypes in *Daam1^gt/gt^* embryos was very low, we analyzed *loop-tail* (*Vangl2^Lp/Lp^*) embryos, which are null mutants for *Vangl2*. In *Vangl2^Lp/Lp^* embryos, both cell morphology and polarity were disrupted, with a large proportion of rounded cells in the optic vesicle (Fig. 4O) and alterations in the distribution of ZO1 and actin (Fig. 4T,W). The Wnt-PCP pathway modulates the cytoskeleton by activating small GTPases, principally RhoA, which leads to the formation of the contractile actin that drives cell polarization (reviewed by Amano et al., 1997; Bishop and Hall, 2000; Klein and Mlodzik, 2005). We decided to test whether inhibition of RhoA or actin microfilaments could recapitulate the eye defects that were observed in embryos exposed to a high concentration of glucose. To this end, embryos were exposed to cytochalasin D, a potent inhibitor of actin microfilament polymerization, and the ROCK inhibitor Y-27632. Both inhibitors caused malformed optic vesicles in cultured embryos. These optic vesicles had round cells that were unpolarized, as evident by β-catenin immunostaining and the perturbed distribution of ZO1, similar to the phenotype observed after exposure to a high concentration of glucose and in the *Vangl2^Lp/Lp^* mice (Fig. 4N–Q,S–V).

To further evaluate the extent of the changes in cell shape observed under the conditions described, we measured cell circularity. There was a significant increase in the circularity of the optic vesicle cells in embryos that had been treated with high levels of glucose (0.71±0.049, *P*<0.0001; where a perfect circle=1) when compared with control embryos (0.49±0.048, *P*<0.0001), as well as in *Vangl2^Lp/Lp^* embryos (0.65±0.05, *P*<0.0001) and in those that had been exposed to cytochalasin D (0.78±0.041, *P*<0.0001) or the Rho inhibitor, Y27632 (0.78±0.048, *P*<0.0001; Fig. 4X).

### *Daam1* mutation and exposure to high levels of glucose reduce the expression of the early eye marker Pax6

**Fig. 5.**
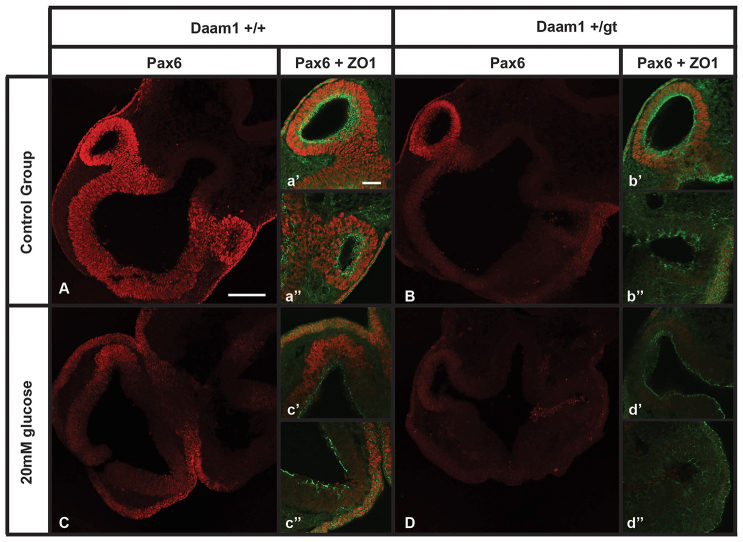
**Pax6 expression in the developing eye in embryos exposed to glucose *in vitro*.** Embryos at E9 obtained from four independent litters were cultured under control conditions (*n*=11; A,B) or in the presence of 20 mM of glucose (*n*=15; C,D). Maximum intensity projections of confocal immunostaining images of Pax6 (red) expression in 50-μm transverse cryosections of *Daam1^+/+^* (A,C); *Daam1^+/gt^* (B,D). Expression of Pax6 and ZO1 (green) on a single confocal section showing in detail the immunostaining in each of the optic vesicles (a′–d′ and a″–d″). Scale bars: 1000 μm (A, also applies to B,C,D); 100 μm (a′, also applies to a″, b′, b″, c′, c″, d′ and d″). All images were taken using the same confocal microscopy parameters for a given primary antibody.

We observed strong disturbances in cell morphology and alterations in the Wnt-PCP pathway as a consequence of exposure to a high concentration of glucose, although it remains unclear how such changes could interfere with eye development. Pax6 is transcription factor that plays a crucial role in eye development. *Pax6* deficient mice either lack or develop small eyes (reviewed by Gehring, 2002). To determine whether *Daam1* mutation and exposure to high levels of glucose could affect Pax6 expression, we performed immunohistochemical studies on embryos that had been cultured under control conditions or in the presence of 20 mM of glucose, and only those that developed to between 20 and 24 somites were analyzed. Maximum intensity projections of confocal microscopy images revealed a homogeneous distribution of Pax6 in the entire optic vesicle and in the area of the surface ectoderm that normally gives rise to the lens in the control *Daam1*^+/+^ embryos (*n*=3; Fig. 5A). At higher magnification, a well-structured epithelium was evident with ZO1 evenly distributed on the apical side of the vesicle (Fig. 5a′,a″). However, both *Daam1*^+/gt^ embryos and *Daam1*^+/+^ embryos that had been exposed to high levels of glucose presented a remarkable reduction in the expression of Pax6 in one of the optic vesicles, although Pax6 expression was, interestingly, unaltered in the surface ectoderm (two out of three and one out of four, respectively; Fig. 5B,b′,b″,C,c′,c″). This downregulation of Pax6 expression in the affected optic vesicles was accompanied by an altered distribution of ZO1 (compare Fig. 5b′,c′ with Fig. 5b″,c″). This phenotype was strongly exacerbated when both of these conditions coincided (three out of four embryos), not only resulting in reduced Pax6 expression in both optic vesicles but also, in a more general manner, affecting even the surface ectoderm (Fig. 5D,d′,d″).

## DISCUSSION

Maternal diabetes has been studied extensively owing to its well demonstrated involvement in the development of embryonic defects, especially those affecting the nervous and cardiovascular systems. However, the molecular mechanisms and signalling pathways affected by maternal diabetes remain unclear. Here, we have found that hyperglycaemia during pregnancy induced a high percentage of eye malformations, among other defects, and that signalling via the Wnt-PCP pathway was impaired in these embryos. Interestingly, our genetic analysis identified a similar eye phenotype to that produced by the *Daam1* mutation, a malformation that worsens dramatically in the presence of environmental hyperglycaemia, both *in vivo* and *in vitro*. Taken together, these observations predict a series of events whereby diabetes affects the Wnt-PCP pathway, which is in turn detrimental to eye development.

### Diabetes and eye development

Our results show that environmental hyperglycaemia makes embryos prone to develop eye defects, including microphthalmia or anophthalmia. Of note, eye defects (microphthalmia or anophthalmia) have been previously reported in children of diabetic mothers (Atasay et al., 2002; James et al., 2007), similar phenotypes have been observed in mouse embryos that were exposed to maternal diabetes (Torchinsky et al., 1997; Machado et al., 2001; Oyama et al., 2009).

Here, we observed a variety of eye defects according to the developmental stage analyzed. Cytoskeletal and tissue disorganization was evident in the optic vesicles of early embryos that had been cultured in the presence of high levels of glucose, whereas the *in vivo* study of older embryos identified a wider optic stalk, an underdeveloped retina and the absence of a lens. In mice, the optic vesicle originates as a bilateral evagination from the diencephalon, which progressively grows towards the surface ectoderm before establishing mutual interactions that lead to a thickening of the overlying surface ectoderm, the ‘lens placode’. During this process, the lumen of the distal optic vesicle widens, whereas the optic stalk narrows. Subsequently, the optic vesicle invaginates and forms the optic cup, under the influence of the lens placode. We believe that exposure to high concentrations of glucose mainly affects the step prior to the initial contact between the distal optic vesicle and the overlying surface ectoderm. If the optic vesicle does not evaginate properly from the telencephalon, changing its shape and contacting the surface ectoderm, the development of the optic cup and the lens will be impaired, provoking the phenotypes observed in our *in vivo* experiments.

### Diabetes and the Wnt-PCP pathway

As mentioned above, diabetes during pregnancy deregulates genes in different pathways, some of which are directly involved in eye development, such as those in the Wnt-β-catenin pathway or the Wnt-PCP pathway. Thus, it is tempting to suggest that the effects on these pathways could be the cause of the eye defects observed in the offspring of diabetic dams. We corroborated the diabetes-mediated impairment of the Wnt-PCP pathway by also analyzing the heart and the neural tube, the development of which is Wnt-PCP dependent. During heart formation, the disruption of PCP signalling results in a range of cardiovascular anomalies, including misalignment of the great arteries with the ventricular chambers, ventricular septation and maturation of the ventricular myocardium, as evident in the PCP mutants *loop-tail* (mutant for *Vangl2*), *Crash* (mutant for *Clsr1*), and the *Dsh* and *Daam1* mutants (Hamblet et al., 2002; Phillips et al., 2005; Phillips et al., 2007; Li et al., 2011; reviewed by Henderson and Chaudhry, 2011). Moreover, there is now evidence that disruption of the Wnt-PCP pathway can cause heart defects in humans (Dauber et al., 2013; Palomino Doza et al., 2013). The *Daam1^+/+^* and *Daam1^+/gt^* embryos that were exposed to diabetes and studied here developed different heart defects, although the most common one was the misalignment of the great arteries, leading to DORV (no such heart defects were evident in the control embryos). The development of DORV in *Daam1^+/gt^* embryos from diabetic dams demonstrates the interaction between diabetes and the Wnt-PCP pathway, because this defect has been observed only in *Daam1^gt/gt^* embryos (Li et al., 2011).

NTDs are related to diabetes, and it has been suggested that alteration of the Wnt-PCP pathway might have implications in the diabetic embryopathies (Pavlinkova et al., 2008). However, we did not detect NTDs in association with diabetes, although interestingly, the only embryo that developed craniorachischisis (a characteristic phenotype of Wnt-PCP mutants) was a *Daam1^+/gt^* embryo that had been exposed to glucose, leading us to question whether this might have been provoked by the interaction between diabetes and the disruption of the Wnt-PCP pathway during neural tube development. Other NTDs were also observed, albeit less frequently than in previous studies, and they were mainly related to diabetes. The C57Bl/6 mouse strain is known to be resistant to the development of NTDs that are a consequence of maternal diabetes (Pani et al., 2002), probably explaining the lower incidence of such defects. Further studies using other mouse strains that are more prone to diabetes-induced NTDs should serve to clarify this issue.

### The Wnt-PCP pathway and eye development

Studies in Xenopus, zebrafish and medaka suggest that the Wnt-PCP pathway might be necessary during the formation and maintenance of the eye field, mediating morphogenetic movements. In zebrafish, ectopic Wnt11 (a Wnt-PCP pathway ligand) induces ectopic expression of Pax6 and the formation of bigger eyes, whereas, when mutated (silberblick mutation) there is a partial fusion of the eye field, possibly reflecting defects in morphogenetic movements (Cavodeassi et al., 2005; Heisenberg et al., 2000). In addition, by using morpholinos in Xenopus it has been demonstrated that the downstream members of the Wnt-PCP pathway, such as Dsh or Daam1, and crosstalk with ephrinB1, are necessary to control the movement of retinal progenitors in the eye field (Lee et al., 2006). Similarly, Xenopus Wnt4 (another ligand for the Wnt-PCP pathway) is expressed adjacent to the eye field, and its downregulation leads to the specific loss of the eye field markers Rx and Pax6 (Maurus et al., 2005). Fzd3, a candidate Wnt4 receptor, appears to be sufficient and necessary for eye formation in Xenopus, the overexpression of which induces Pax6 expression (Rasmussen et al., 2001). By contrast, in mice, there is no clear evidence that the Wnt-PCP pathway participates in early eye development, although lens cell alignment is altered in mice carrying mutations in *Vangl2* and *Celsr1*, affecting the final lens morphology (Sugiyama et al., 2010). However, the incidence of microphthalmia and anophthalmia observed as a consequence of *Daam1* downregulation, together with the early detection of some Wnt-PCP genes in the main eye structures (e.g. the perioptic plexus, the optic cup, the optic nerve or the lens), strongly implicates this pathway in eye development.

Here, we show that the cells in the optic vesicle are dysmorphic in embryos with a disrupted Wnt-PCP pathway, having an altered polarity and a rounder shape. In addition to the gene expression analysis and the interaction between the *Daam1* mutation and diabetes in eye development, a number of morphological and apicobasal cell polarity defects were found in the optic vesicles of embryos that had been exposed to glucose. These cellular defects were associated with altered actin, myosin and ZO1 distributions. Similar results were obtained when the optic vesicles of a Wnt-PCP mutant, *loop-tail*, were analyzed, strongly implicating the Wnt-PCP pathway in the establishment of the appropriate cell settings for the early steps of eye development. Moreover, the Wnt-PCP pathway is directly associated with the Rho-ROCK system, and disruption of ROCK signalling exacerbates the NTDs in mice carrying mutations in the Wnt-PCP pathway. We found that, by blocking an intermediate step in the pathway, a direct effector of RhoA (ROCK), we obtained optic vesicles that phenocopied those observed after treatment with glucose in culture and in the *Vangl2^Lp/Lp^* embryos. In a very neat study using embryonic stem-cell-derived neuroepithelial vesicles, the process of optic cup formation could be mimicked in the mouse embryo, dividing it into four phases (Eiraku et al., 2011). The first three phases involved (i) evagination of the columnar monolayered epithelial cells to form a vesicle (phase 1); (ii) flattening of the distal portion of the vesicle (phase 2); and (iii) adjustment of the angle at the joint between the neural retina and the retinal pigmented epithelium through cell wedging and bending (phase 3). The regulation of this latter phase by the Rho-ROCK system is consistent with our observations.

Microfilaments are key internal regulators of epithelial morphogenesis, and they are often regulated by the Rho-ROCK system (reviewed in Sawyer et al., 2010). In the neural tube, Rho predominantly accumulates near the apical region of the neuroepithelium, overlapping with actin filaments, a process which was thought to demonstrate the involvement of Rho in apical constriction and concomitant cytoskeletal reorganization. Similarly, blocking actin polymerization in our system produces similar results in the developing optic vesicles as those obtained after inhibition of Rock. Indeed, perturbed cell shape and cell polarity is a consequence of altering the Wnt-PCP pathway or the Rho-ROCK system in other animal models (Ybot-Gonzalez et al., 2007; Gray et al., 2013).

We also found that the cellular defects observed in the optic vesicle in embryos *Daam1*^+/gt^ and in those treated with glucose, were accompanied by reduced Pax6 expression in the optic vesicle. Pax6 is crucial in eye development, and mutations in the insect and mammalian Pax6 homologues prevent eye development (reviewed by Gehring, 2002). *Small eye* mice are homozygous for a mutation in the Pax6 gene and are reported to have no lenses, an identical phenotype to that observed in *Daam1^gt/+^* embryos that had been exposed to maternal diabetes. We demonstrate that the Wnt-PCP pathway is downregulated in embryos that are exposed to high levels of glucose, as is Pax6 expression, in a similar manner to the downregulation of Pax6 in the optic vesicle of *Daam1^gt/+^* embryos. The reduction of Pax6 in the optic vesicle could reflect the role of the Wnt-PCP pathway in eye fate determination. For example, loss of Wnt4 function in Xenopus results in a specific loss of Pax6 (Maurus et al., 2005). Hence, the Wnt-PCP pathway appears to be directly implicated in eye fate determination because it controls the expression of specific eye field genes. Conversely, if the optic vesicle relies on a well-structured epithelium and on specific cell movements induced by the Wnt-PCP pathway in order to preserve its identity, when this developmental process is inhibited, a partial loss of the epithelial character of the optic vesicle could be provoked, along with the loss of specific eye markers, such as Pax6. In this case, the Wnt-PCP pathway would fulfil a more morphogenetic role during eye development. Indeed, because partial inhibition of the Wnt-PCP pathway only affects the expression of Pax6 in the optic vesicle and not in the surface ectoderm, we believe that the developing eye relies on the Wnt-PCP-driven cell movements in the optic vesicle to keep its epithelial status at this developmental stage, which is in turn crucial to achieve its fate. However, further studies will be required to be able to clearly discern between these two possibilities.

In conclusion, we have shown here that the Wnt-PCP pathway is involved in the early steps of eye development (e.g. optic vesicle evagination and optic cup formation) and that maternal diabetes disrupts the Wnt-PCP pathway, leading to the loss of epithelial features and of specific eye field markers. These alterations ultimately might cause distinct embryopathies, including eye defects.

## MATERIALS AND METHODS

### Animals

The *Daam1* gene trap mutant mouse strain was obtained from Baygenomics (cell N° RRT390). This mouse strain carries a mutation that causes incomplete loss of function due to the alternate gene splicing (Li et al., 2011). The *loop-tail (Vangl2^Lp^)* inbred strain, which carries the *Vangl2* mutation, was originally obtained from Jackson Laboratories. The *Daam1* mutation was maintained in a C57BL/6 background, and the *loop-tail* mutant was on a CBA background.

Mice were maintained on a 12-hour light:12-hour dark cycle (lights on from 07:00–19:00), with *ad libitum* access to food (Global Diet 2014S, Harlan-Teklad) and water. After overnight mating, dams were checked for vaginal plugs the following morning, and the day on which a copulation plug was found was designated as E0.5. Litters were collected at intervals between E11.5 and E17.5.

All procedures involving any experimental animals were performed in compliance with local animal welfare laws, guidelines and policies.

### Diabetes

Diabetes was induced using a protocol of multiple low-dose STZ (S0130, Sigma-Aldrich) injections. Female mice were injected intraperitoneally with STZ on five consecutive days at a concentration of 50 mg/kg of body weight. STZ was prepared fresh in 0.1 M sodium citrate buffer (pH 4.5) to a final concentration of 7.5 mg/ml (Tesch and Allen, 2007). Blood glucose levels were monitored 10 days after the last STZ injection using a commercial glucometer (Accu-Chek Aviva Nano, Roche Diagnostics). Mice with non-fasting blood glucose above 250 mg/dl (Pavlinkova et al., 2008) were considerer diabetic mice. Diabetic and age-matched control female mice were mated with *Daam1^+/gt^* male mice.

### General morphology and histological analysis of embryos and foetuses

Embryos were collected at E10.5-17, and their membranes were used for genotyping through conventional PCR analyses using specific *Daam1* (Li et al., 2011) and *loop-tail* primers (Stanier et al., 1995). Foetuses obtained between E12 and E17 were analyzed for external malformations and photographed by using a stereomicroscope (SteREO Discovery V8+AxioCam Erc8, Zeiss). After fixation with 4% paraformaldehyde (PFA) for 1 hour, the embryos were stored in 70% ethanol, serially dehydrated and embedded in paraffin wax. Microtome sections (7 μm; Leica RM2255) from at least three different embryos were stained with Ehrlich’s haematoxylin and eosin for histological analysis and mounted with Hydromount (HS-106, National Diagnostics). Representative sections were selected and photographed by using an Olympus (Tokyo, Japan) BX-61 photomicroscope.

### *In-situ* hybridization and X-gal staining

To study mRNA expression, whole mount *in situ* hybridization was performed using sense and antisense riboprobes prepared with a digoxigenin RNA labelling kit (Roche, Basel, Switzerland) according to the manufacturer’s instructions. At least three mouse E9.5 embryos (25–30 somites) were analyzed per probe, as described previously (Ybot-Gonzalez et al., 2005). Selected embryos, labelled for *Daam1* (Ybot-Gonzalez et al., 2007), glypican4 (Ybot-Gonzalez et al., 2005) or *Wnt5a* (Gavin et al., 1990) were embedded in a gelatin-sucrose-albumin and glutaraldehyde solution, and vibratome sections (50 μm) of the embryos were photographed by using an Axiophot (Zeiss, Jena, Germany) photomicroscope. To compare *Daam1* expression between control and treated embryos, the *in situ* hybridization procedure was performed in only one tube, adequately marking the embryos for their later identification. Sense-strand control riboprobes gave no specific signal.

Embryos at E11.5 and E12.5 were fixed in 4% PFA, and they were immediately whole mount stained using the X-Gal method (Carroll et al., 2003). Eosin counterstained paraffin sections (7 μm) were examined.

### Immunohistochemistry

For immunohistochemical analysis, embryos at E9.5 were embedded in gelatin (15% sucrose and 7.5% gelatin in PBS) and cryo-sectioned at thicknesses of 10 μm and 50 μm on a Leica CM1950 cryostat. Sections of at least three different embryos per primary antibody were blocked with PBT (PBS, 10% Triton and 1% BSA) and incubated overnight at 4°C with the primary antibody against myosin heavy chain II-B (PRB-445P, Covance) diluted 1:150 in PBT; β-catenin (610153, BD Biosciences) diluted in PBT 1:200; ZO1 (40-2200, Invitrogen) diluted 1:100 in PBT; or Vangl2 (a kind gift from Mireille Montcouquiol, Neurocentre Magendie, Bordeaux, France) (Belotti et al., 2012) diluted 0.5:150 in PBS and 5% BSA; or Pax6 [mouse monoclonal, Developmental Studies Hybridoma Bank (DSHB)] diluted in PBT 1:200. The secondary antibodies used were as follows: goat anti-rabbit FITC-conjugated for the primary antibodies against myosin II, ZO1 and Vangl2 (ab6717, Abcam) or a Cy™3-conjugated affiniPure goat anti-mouse IgG to detect antibodies staining β-catenin and Pax6 (115-165-166, Jackson ImmunoResearch). Phalloidin-Tetramethylrhodamine B isothiocyanate was used to visualize F-actin (P1951, Sigma-Aldrich). Sections were mounted with Hydromount (HS-106, National Diagnostics), and then photographed by using an Olympus (Tokyo, Japan) BX61 microscope or a confocal microscope (TCS-SP2-AOBS, Leica).

### Embryo culture

Embryos at E9, with their embryonic membranes intact, were cultured in the presence of D-glucose dissolved in PBS (10 mM, 20 mM or 30 mM), or with cytochalasin D (CytD, 0.05 μg/ml, 1233, Tocris Bioscience) and Y-27632 (10 μM, 688000; Calbiochem). Embryos were maintained at 37°C in culture for 7 hours (in the presence of glucose) or 6 hours (for the CytD and Y-27632 treatment), as described previously (Copp et al., 2000). In each experiment, control embryos were cultured in parallel in the presence of the vehicle solution alone. After culture yolk sac circulation and the heart beat of the embryos had been assessed, only apparently healthy embryos were studied further to assess external malformations and subsequently fixed in 4% PFA.

### RNA extraction and quantitative RT-PCR

The expression of genes in the Wnt-PCP pathway was analyzed by using quantitative RT-PCR. Cultured 22- to 23-somite embryos were preserved in RNA-later (Ambion) until total RNA was extracted with the TRIsure reagent (Bioline) according to the manufacturer’s recommendations. The One-Step SYBR PrimeScript RT-PCR kit (TaKAra, Otsu, Shiga, Japan) was used to synthesize first-strand cDNA from 40 ng of the total RNA. Quantitative RT-PCR analyses were performed using the ViiA7 Real-Time PCR system (Applied Biosystems). The primer set used for quantitative RT-PCR analyses of *Daam1* was validated. The primers were: *Daam1* (F) 5′-GAAGAGTTGCGGGATATTCC-3′ and (R) 5′-TCTGAGAAGGTGAAGCTAGC-3′. The primers used against *Vangl2* were those described previously (Chen et al., 2013). The primer set for the internal control, *Hmbs* (Hidalgo-Figueroa et al., 2012), was (F) 5′-CCATACTACCTCCTGGCTTTACTATTG-3′ and (R) 5′-GGTTTTCCCGTTTGCAGATG-3′. *Hmbs* was expressed stably under the experimental conditions used. The reactions were run in triplicate and repeated on several independent samples for each genotype. The fold change in gene expression was determined using the Ct method, normalizing expression to the housekeeping *Hmbs* target gene and to a control untreated sample.

### Estimation of cell circularity

When assessing cell polarity, it is useful to define to what extent the width and length of the cell approximates to a circle (circularity). The number of apical surface cells in the optic vesicle in each condition were: control embryos, 220 cells, 6×50 μm cryosections from four embryos of two different litters; 20 mM glucose, 100 cells, 10×10 μm cryosections from five embryos of two different litters; cytochalasin D, 280 cells, 5×50 μm cryosections from five embryos of two different litters; Y-27632, 150 cells, 4×50 μm cryosections from four embryos of two different litters; and *Vangl2^Lp/Lp^* mutant embryos, 150 cells, 3×50 μm cryosections from three embryos of two different litters. At least ten cells at the apical surface of the optic vesicle were analyzed per cryosection from each embryo. The area and the perimeter of the cells were measured using ImageJ software (National Institutes of Health), and circularity was determined using the equation: circularity=4π (area/perimeter^2^).

### Statistical analysis

Categorical variables, including the number of affected embryos from diabetic dams and the number of affected *Daam1* mutants, were evaluated using a Fisher’s exact test. Quantitative variables, including litter size, the number of resorptions and the fold-change values were evaluated using a U-Mann–Whitney test. Global comparisons for cell circularity were evaluated using the H-Kruskal–Wallis non-parametric test. Multiple comparisons for cell circularity analysis were evaluated using a U-Mann–Whitney test attending to the Bonferroni correction (*P*<0.0125). The software used for statistical analysis was IBM PSS Statistics 19.

## Supplementary Material

Supplementary Material
