# Supplementary material for: The effect of maternal diabetes on the Wnt-PCP pathway during embryogenesis as reflected in the developing mouse eye
Source: Dis Model Mech. 2014 Dec 24;8(2):157–68. doi: 10.1242/dmm.017723 (PMC4314781; doi:10.1242/dmm.017723)
Supplement: Supplementary Material [file supp_8_2_157__index.html]

The effect of maternal diabetes on the Wnt-PCP pathway during embryogenesis as reflected in the developing mouse eye — Supplementary Material 

# The effect of maternal diabetes on the Wnt-PCP pathway during embryogenesis as reflected in the developing mouse eye

## DMM017723 Supplementary Material

**Files in this Data Supplement:**

- **Supplementary Material**
