# Supplementary material for: The effect of maternal diabetes on the Wnt-PCP pathway during embryogenesis as reflected in the developing mouse eye
Source: Dis Model Mech. 2014 Dec 24;8(2):157–68. doi: 10.1242/dmm.017723 (PMC4314781; doi:10.1242/dmm.017723)
Supplement: Supplementary Material [file supp_8.2.157_DMM017723.pdf]

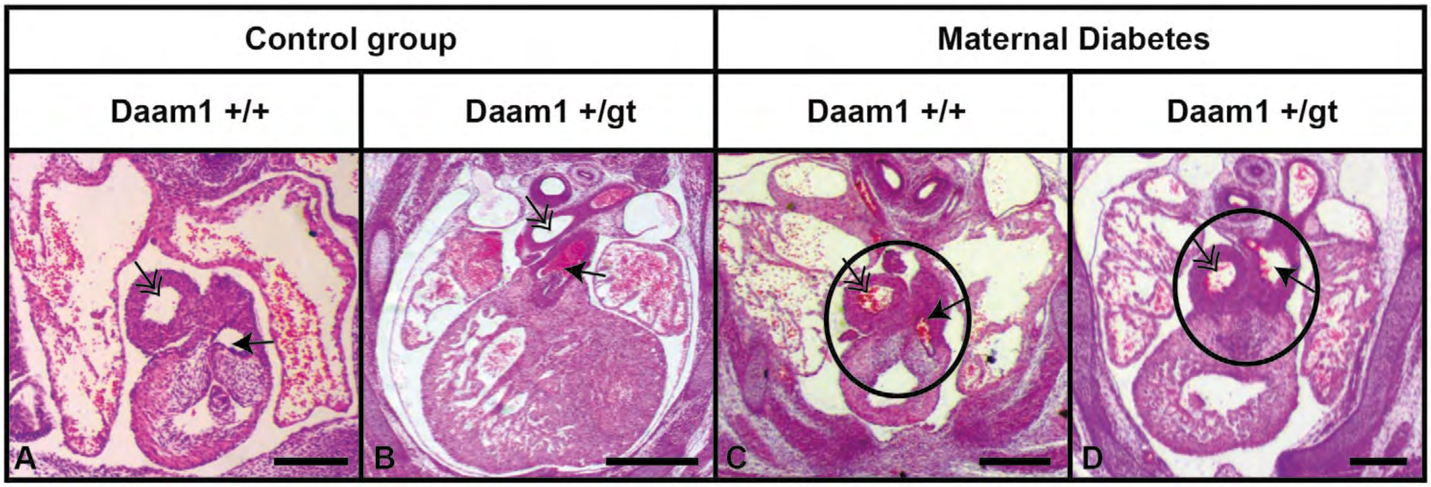


**Fig. S1. Cardiac defects produced by Daam1 knockdown in combination with diabetes**. Transverse haematoxylin and eosin stained paraffin section of E13-E14 embryos at the level of the heart. $Daam1^{+/+}$ and $Daam1^{+/gt}$ from control dam (A and B respectively), $Daam1^{+/+}$ and $Daam1^{+/gt}$ embryos exposed to maternal diabetes (C and D respectively). The main cardiac features are indicated: DORV (encircled in a solid line, panels C and D); the aortic trunk (double arrow) and pulmonary trunk (arrow). Scale bar A: 200 µm; Scale bar B: 500 µm; Scale bar C and D: 250 µm.

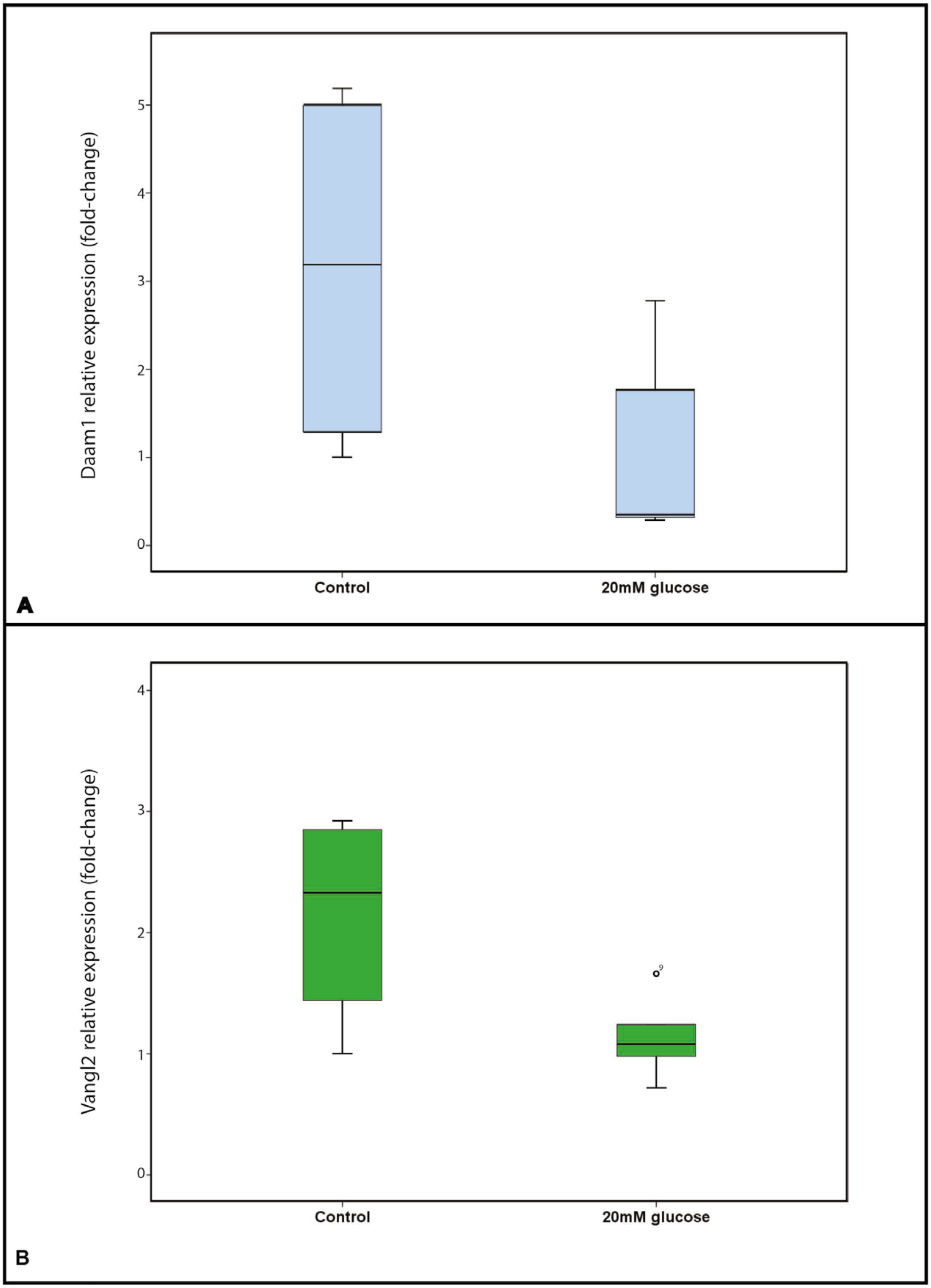


**Fig. S2. Expression of *Daam1* and *Vangl2* in embryos exposed to glucose *in vitro*.** Quantitative RT-PCR for *Daam1* (A) and *Vangl2* (B) normalized to expression levels of *Hmbs* in E9 embryos after culture in absence (control) and presence of glucose (20mM glucose). No statistically significantly differences in the expression levels were obtained (*Daam1* $P$=0.142; *Vangl2* $P$=0.086). Error bars indicate standard deviation.
